# Small cell neuroendocrine carcinoma in maxillary sinus: a case report and literature review

**DOI:** 10.1016/j.ijscr.2024.110321

**Published:** 2024-09-19

**Authors:** Hugo Humberto Romero Alvarenga, Juan José Guifarro, Francisco Díaz, Agatha Reyes, Jean Luis Piedra Burneo

**Affiliations:** a Department of Oral and Maxillofacial Surgery of “Hospital Escuela Universitario de Honduras”, Honduras; b Medical Oncologist at the Hemato-Oncology Unit of the Roosevelt Hospital, Guatemala; c Postgraduate degree in oral and maxillofacial surgery, National Autonomous University of Mexico, Mexico City, Mexico

**Keywords:** Carcinoma, Small cell, Therapy, Neuroendocrine tumors

## Abstract

**Introduction:**

Small cell neuroendocrine carcinoma is a rare and aggressive pathology with significant diagnostic challenges. Treatment typically involves multimodal therapy, including surgery and chemotherapy, but outcomes vary. The objective of this study is to describe and report a case of small cell neuroendocrine carcinoma.

**Case presentation:**

A case study illustrates the follow-up of a 36-year-old female patient from diagnostic biopsy to maxillectomy, followed by adjuvant chemotherapy with cisplatin and etoposide. Small cell neuroendocrine carcinoma poses significant challenges due to its rarity and aggressive behavior.

**Discussion:**

Multimodal therapy remains the mainstay, but the prognosis is unfavorable. Despite advances, managing small cell neuroendocrine carcinoma remains challenging.

**Conclusions:**

Integrated approaches are crucial, underscoring the need for ongoing research to improve outcomes in this rare malignancy.

## Highlights

• Surgical intervention combined with radiotherapy or chemotherapy, demonstrated favorable outcomes for Small Cell Neuroendocrine Carcinoma.
• Small Cell Neuroendocrine Carcinoma, can be notably more aggressive than other neuroendocrine types and has the potential to metastasize distantly.
• Cisplatin is an effective and viable option for treating Small Cell Neuroendocrine Carcinoma of head and neck.

## 1 Introduction

Neuroendocrine tumors are a diverse group of neoplasms originating from the diffuse endocrine system. They were first identified in 1907 in the small intestine and were initially termed “carcinoid tumors” due to their benign appearance, despite exhibiting cancer-like characteristics such as the ability to cause local recurrence and metastasis. In 1965, Chowdhuri et al. [1] described the first neuroendocrine carcinoma in the sinonasal tract, and in 1969, Goldman et al. reported a carcinoid tumor in the larynx. As research progressed, it became evident that these tumors differed in their histopathological features and clinical behavior, leading to the establishment of subtype classifications [2,3,4] (Table 1).

**Table 1:**
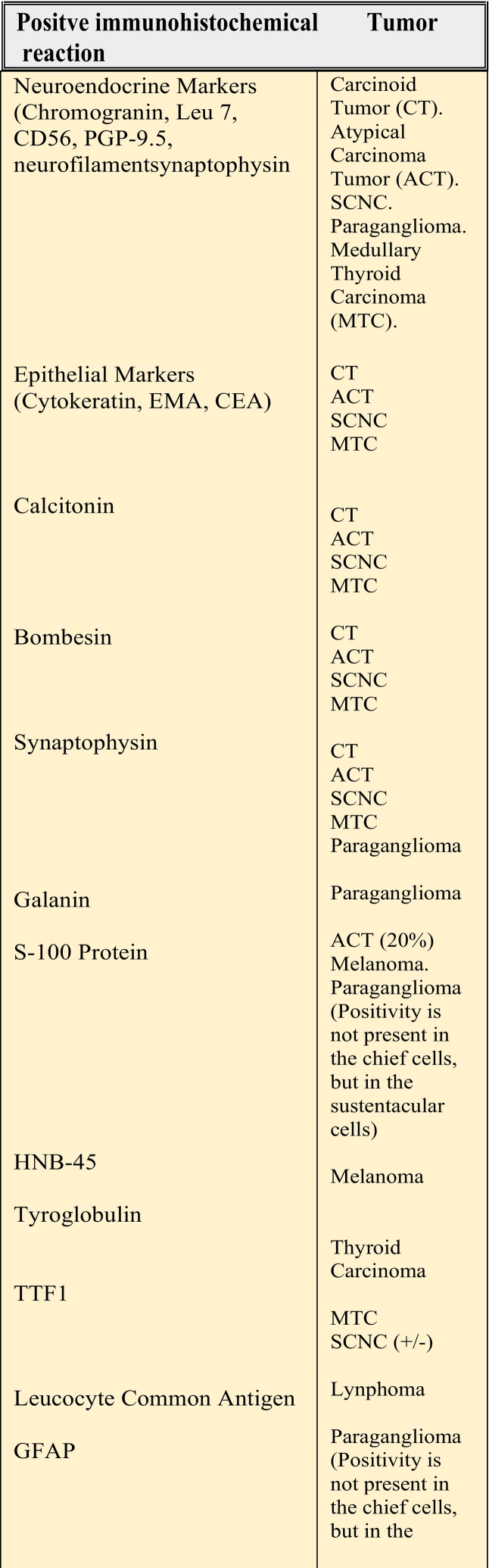
Inmunohistochemical featus in specific differencial diagnosis of different tumors.

**Fig. 1:**
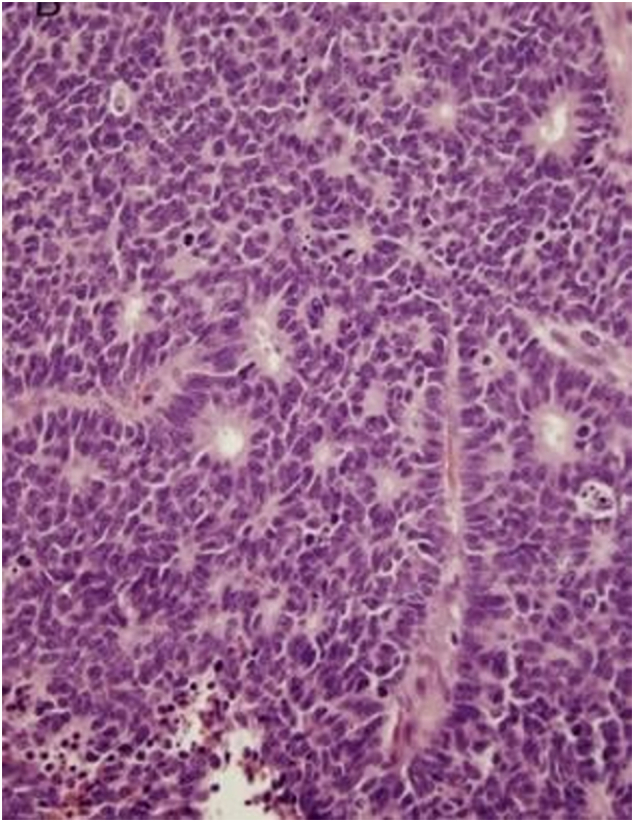
Histological view. The tumor was composed of small round cells of round to fusiform shape, with scanty cytoplasm, fine granular.

Neuroendocrine tumors (NETs) originate from cells that are part of both the endocrine and nervous systems. They are thought to arise from APUD cells (amine precursor uptake and decarboxylation), which are associated with the endocrine system but produce hormones from epithelia rather than glands. These cells are occasionally found in accessory or salivary glands within the nasal cavity. NETs can be either benign or malignant and may develop in various parts of the body, typically corresponding to the distribution of their precursor cells, such as Kulchitsky cells or similar enterochromaffin-like cells (Fig. 1) [5,6,7]. Common sites of occurrence include gastrointestinal tract (67 %), lungs (22 %) the pituitary, adrenal, thyroid, and parathyroid glands, as well as the thymus, breast, genitourinary tract, peripheral nervous system, and skin. However, NETs can also arise in locations lacking their typical precursor cells, such as the larynx and sinonasal tract (4 %) [8,9,10] (Table 2).

**Table 2:**
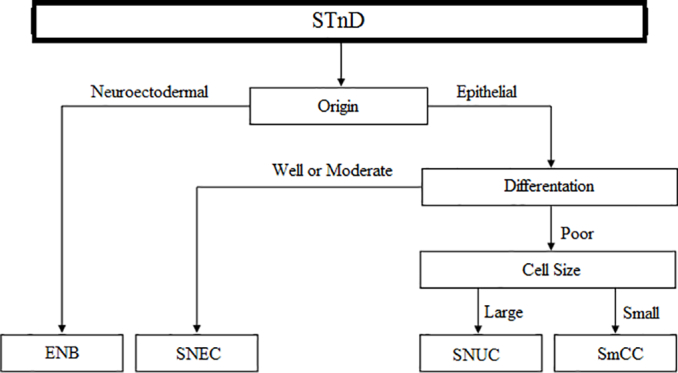
Classification of sinonasal tumors with neuroendocrine differentiation. STND, Sinonasal Tumors with Neuroendocrine Differentiation; ENB, Esthesioneuroblastoma; SNEC, Sinonasal Neuroendocrine Carcinoma; SNUC, Sinonasal Undifferentiated Carcinoma; SmCC, Small Cell neuroendocrine Carcinoma. van der Laan, T. P. (2017). Neuroendocrine carcinoma of the head and neck: in search for a betteroutcome. [Groningen]: Rijksuniversiteit Groningen.

According to the World Health Organization (WHO) classification, NETs are categorized into: (a) typical carcinoid, (b) atypical carcinoid, (c) small cell carcinoma, neuroendocrine type, (d) combined small cell carcinoma, neuroendocrine type, and (e) paraganglioma, which can be benign or malignant. Extrapulmonary small cell neuroendocrine carcinoma (SNEC) is relatively rare, with the head and neck region—particularly the larynx and salivary glands—being common sites of origin [5,11,12,13].

### 1.1 Case report

**Fig. 2:**
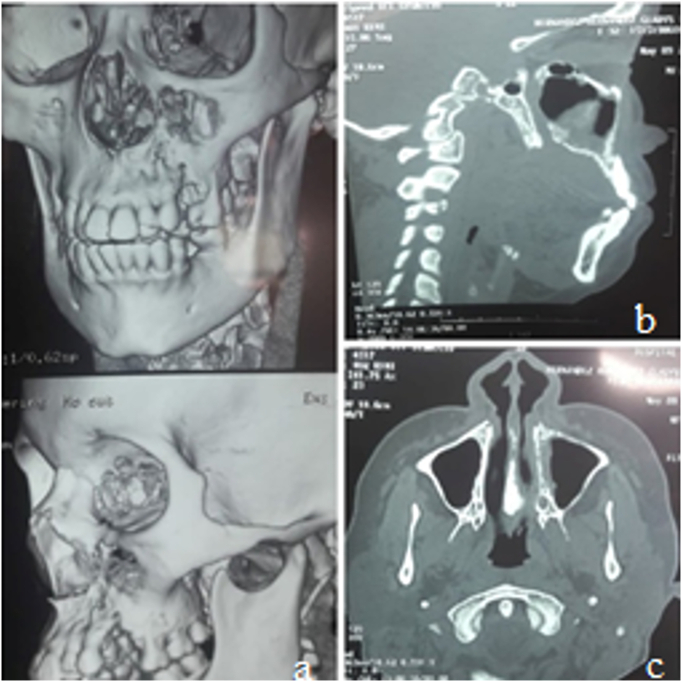
Shows the volumetric reconstruction of the tomography. it is possible to observe a dehiscence of the anterior wall of the maxillary sinus, due to tumor growth. Figure “b” shows a sagittal cut, where it is possible to observe an isodense area within the maxillary sinus. Figure “c” shows an axial view, where an isodense zone is observed that crosses the medial wall of the maxillary sinus.

This report presents the case of a 36-year-old female diagnosed with small cell neuroendocrine carcinoma (SNEC). She was presented to the maxillofacial surgery department with a tumor in the maxillary region and pain persisting for four months. Radiographic examinations, specifically computed tomography (CT), revealed an isodense area in the maxillary sinus region (Fig. 2). This report was documented in accordance with the SCARE criteria.

Before proceeding with definitive treatment, an incisional biopsy of the lesion was performed, confirming the diagnosis of small cell neuroendocrine carcinoma. The treatment plan involved maxillary resection.

### 1.2 Surgical notes

**Fig. 3:**
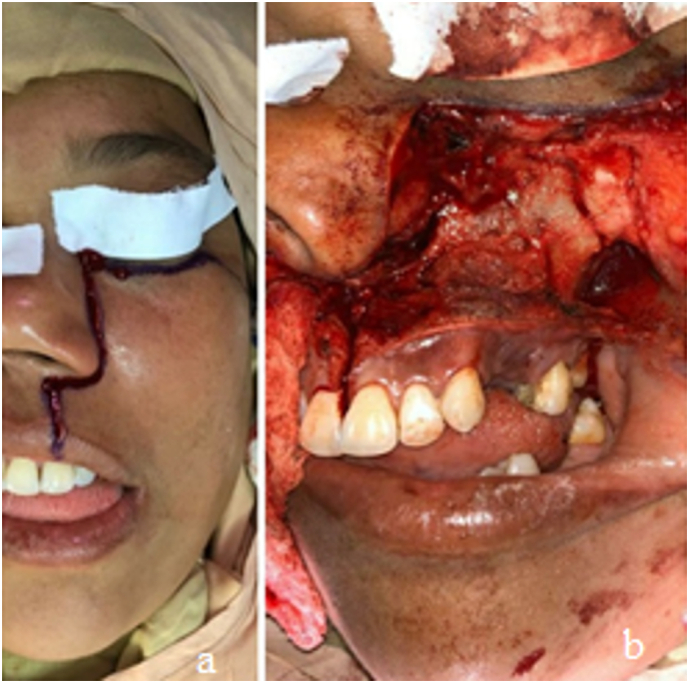
Shows the design of the Weber-Ferguson-type approach. Figure b shows the displaced flap and the surgical area.

A maxillectomy (Brown classification: 2B) was performed on the left maxilla under general anesthesia. Asepsis and antisepsis were maintained using povidone‑iodine, and a pharyngeal pack was placed. Local anesthesia (lidocaine 2 % with epinephrine 1:100,000) was administered, and a Weber-Ferguson-type incision was made (Fig. 3).

**Fig. 4:**
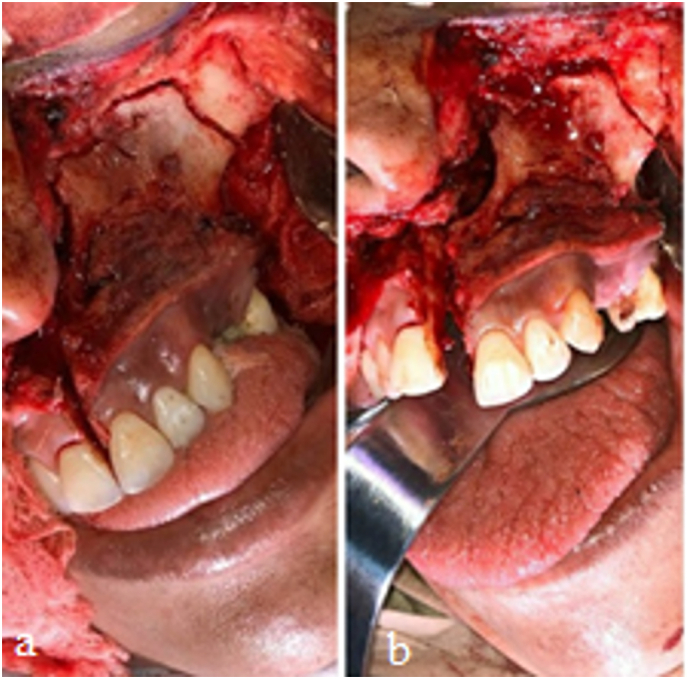
Shows the cut in the maxilla for resection. Figure b shows the dislocation of the maxillary portion to be removed.

**Fig. 5:**
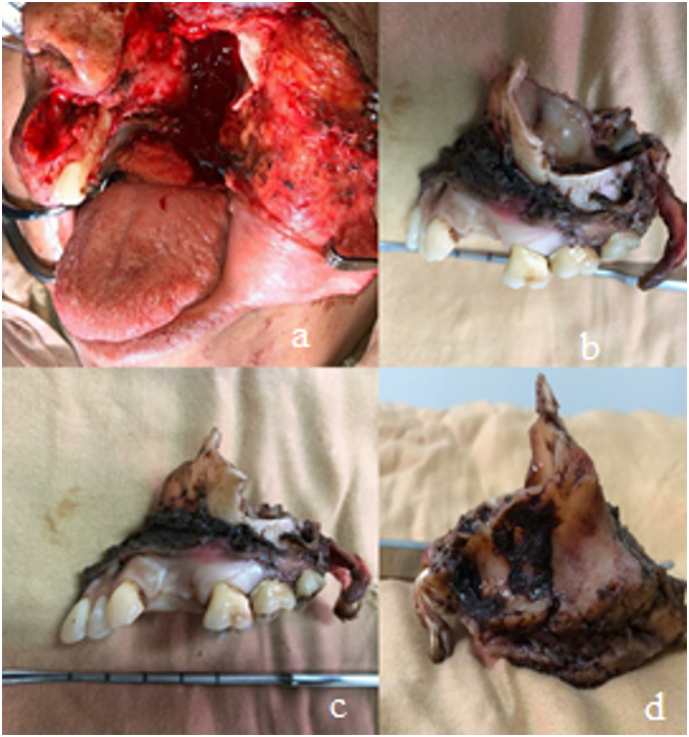
Shows the surgical area after resection of the maxilla. Figures b, c and d show the maxilla after being removed.

The bone cuts were made using a reciprocating saw, and the maxilla was resected. The flap was then repositioned, and the appropriate sutures were placed. The procedure was completed without complications (Fig. 4, Fig. 5).

### 1.3 Immunohistochemical analysis

**Fig. 6:**
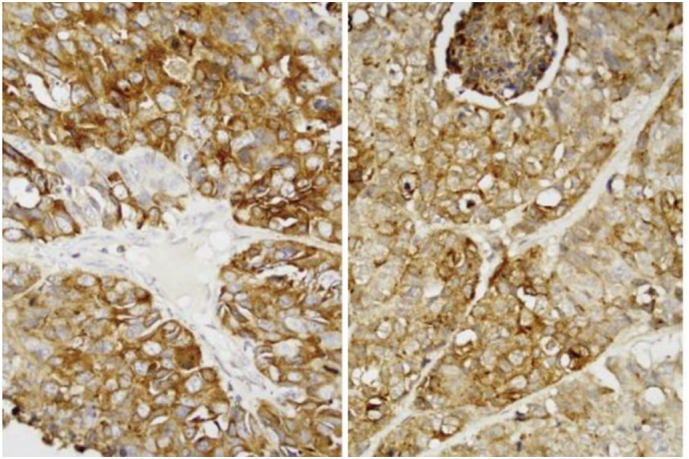
Immunohistochemical analysis was requested and the results showed positivity for the antibodies CD10, SYN, chomogranin, with a strong staining for Ki67, and focal staining for vimentin. The CK20, cytokeratin AE1/AE3, PSA, and CK7 antibodies were negative. Histological view show the tumor was composed of small round cells of round to fusiform shape, with scanty cytoplasm, fine granular.

Immunohistochemical analysis showed positivity for CD10, synaptophysin (SYN), and chromogranin, with strong staining for Ki-67 and focal staining for vimentin. Antibodies for CK20, cytokeratin AE1/AE3, PSA, and CK7 were negative. The histological examination revealed that the tumor was composed of small, round cells with round to fusiform shapes, scant cytoplasm, and fine granular chromatin (Fig. 6).

### 1.4 Chemotherapy phase

The chemotherapy regimen included cisplatin at 45 mg/m^2^/day (administered intravenously as a continuous infusion) on days 1–3, and etoposide at 130 mg/m^2^/day (administered intravenously as a continuous infusion) on days 1–3, for a total of six cycles.

**Fig. 7:**
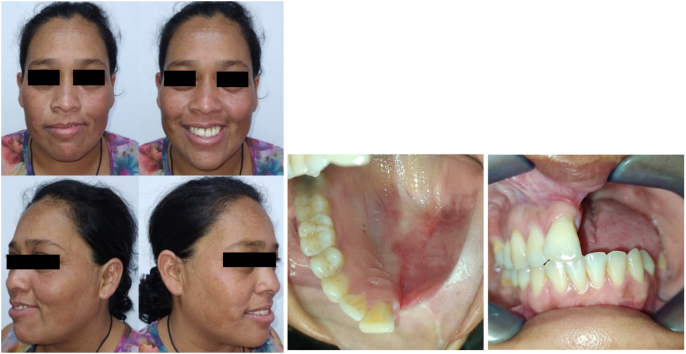
Postoperative extraoral and intraoral photograph of three year of evolution.

Three years postoperatively, the patient was reassessed clinically, showing satisfactory results (Fig. 7).

## 2 Discussion

Small cell neuroendocrine carcinomas (SNECs) are an exceptionally rare type of tumor, and there is limited clinical experience regarding their management. When occurring outside the lungs, they most commonly arise in the larynx and paranasal sinuses within the head and neck región [14]. The first case of SNEC was reported by Raychowdhuri in 1965, and since then, only a few cases have been documented in the literature [15]. Anastasia F. et al. [16] note that carcinoma of the nasal cavity and paranasal sinus is particularly uncommon, with an annual incidence of <1 per 100,000 individuals, accounting for just 3 % of all head and neck malignancies.

It is important to establish an accurate diagnosis using both histopathology and immunohistochemistry. According to Rivero et al. [17], the most common positive immunohistochemical markers include chromogranin, neuron-specific enolase, cytokeratin, and synaptophysin. In this case, chromogranin was the reported positive marker. Approximately 10 % of patients with small cell neuroendocrine carcinoma (SCNEC) develop a syndrome of inappropriate secretion of antidiuretic hormone (SIADH); however, this is even rarer in SCNEC of the head and neck. In our patient's case, this syndrome did not occur.

The modalities of treatment for SNEC are surgery, radiotherapy and chemotherapy, Han et al. [15] refer that the survival of SNEC is 12 months, with a recurrence of 43 %. Perez et al. [18] refer that SNEC had a recurrence or died in 37 months. Patients with Extrapulmonary SCNEC (EP-SCNEC) have a notably grim prognosis, with a 5-year survival rate of 13 % and a median survival from diagnosis of 14.5 months, though survival outcomes partly hinge on the primary tumor site [8,17]. surgical intervention combined with radiotherapy or chemotherapy, demonstrated favorable outcomes for SCNEC [8,[18], [19], [20]]. Babin et al. in their study refer that treatment strategy with platinum-based chemotherapy and radiotherapy when the surgical intervention involves extensive resection [11]. The dissection of lymph node is not justified. In a therapeutic protocol they use cisplatin 33 mg/m2/day for 3 days and Etoposide 100 mg/m2/day) for 3 days if the response is more or <50 %, they decide to use surgery or radiotherapy [11]. Kenmotsu et al. [21] refer that the use of irinotecan and cisplatin was feasible and active in patients with resected HGNEC; generally, between 3 and 4 cycles were used [[22], [23], [24], [25], [26], [27], [28]].

## 3 Conclusions

Small cell neuroendocrine carcinoma is a malignant epithelial tumor most commonly found in lung tissue, with occurrences in other sites, such as the maxillary sinus or paranasal sinuses, being exceedingly rare. This tumor is highly aggressive and poses a significant threat to life.

Treatment strategies for neuroendocrine tumors vary based on their histological characteristics. For small cell carcinoma, a combination of surgery and chemotherapy has proven to be an effective approach. This tumor subtype is often more aggressive than other neuroendocrine types and has a higher potential for distant metastasis, necessitating a proactive treatment strategy. Radical surgery, along with chemotherapy, has shown efficacy in managing this aggressive tumor subtype.

## Consent

Written informed consent was obtained from the patient for publication and accompanying images. A copy of the written consent is available for review by the Editor-in-Chief of this journal upon request.

## Disclosures

Authors must disclose the use of generative AI and AI-assisted technologies in the writing process of Small Cell Neuroendocrine Carcinoma in Maxillary Sinus A Case Report and Literature Review.

The work Small Cell Neuroendocrine Carcinoma in Maxillary Sinus A Case Report and Literature Review. has not been published previously, it is not under consideration for publication elsewhere, that its publication is approved by all authors and tacitly or explicitly by the responsible authorities where the work was carried out, and that, if accepted, it will not be published elsewhere in the same form, in English or in any other language, including electronically without the written consent of the copyright-holder.

## Ethical approval

According to the guidelines of the Institutional Review Board (IRB), a clinical case does not require ethical review if it is a retrospective analysis of a single patient, does not involve experimental interventions, the information is anonymized or consent has been obtained, the purpose is educational, and there are no additional risks to the patient. These factors ensure that the report is ethically handled without the need for IRB review. PAHO Ethics Review Committee (PAHOERC).

## Funding

This case report was self-funded by the authors.

## Author contribution

Hugo Humberto Romero Alvarenga: Study Concept, Data analiys.

Juan José Guifarro: Study Concept, wrting the paper.

Francisco Díaz: study concept, data analysis.

Agatha Reyes: data collection.

Jean Luis Piedra Burneo: writing the paper, data collection.

## Guarantor

Jean Luis Piedra Burneo.

## Research registration number

1. Name of the registry: N/A.
2. Unique identifying number or registration ID: N/A.
3. Hyperlink to your specific registration (must be publicly accessible and will be checked): N/A.

## Conflict of interest statement

All authors disclaim any potential conflicts of interest.
